# Determinants of excessive weight loss in breastfed full-term newborns at a baby-friendly hospital: a retrospective cohort study

**DOI:** 10.1186/s13006-020-00263-2

**Published:** 2020-03-24

**Authors:** Yasuhiro Miyoshi, Hideyo Suenaga, Mikihiro Aoki, Shigeki Tanaka

**Affiliations:** Department of Pediatrics, National Hospital Organization Nagasaki Medical Center, Kubara 2-1001-1, Omura, Nagasaki, 856-8562 Japan

**Keywords:** Newborn, Breastfeeding, Birth weight, Weight loss, Caesarean section

## Abstract

**Background:**

Excessive weight loss in newborns is associated with neonatal complications such as jaundice and dehydration, which cause renal failure, thrombosis, hypovolemic shock, and seizures. The identification of the risk factors for excessive weight loss will help to discover preventive measures. The aim of this study was to determine the factors associated with excessive weight loss, defined as weight loss of ≥10%, in breastfed full-term newborns in Japan.

**Methods:**

The present retrospective study, which was performed in a tertiary perinatal center accredited as a Baby-Friendly Hospital, included neonates who were born alive with a gestational age of ≥37 weeks. Cases of multiple births, admission to the neonatal intensive care unit (NICU), referral to another facility, or exclusive formula feeding were excluded. Multivariate logistic regression analyses were performed to assess the association between maternal or neonatal characteristics and excessive weight loss.

**Results:**

We studied 399 newborns, of whom 164 (41%) had excessive weight loss. According to the adjusted multiple regression analysis, the factors associated with excessive weight loss were an older maternal age, primiparity, and antepartum Caesarean section, with adjusted odds ratios (95% Confidence Intervals [CIs]) of 1.07 (1.02, 1.11), 2.72 (1.69, 4.38), and 2.00 (1.09, 3.65), respectively.

**Conclusions:**

Close monitoring of infants born to older mothers, primiparous mothers, or infants delivered by antepartum Cesarean section is recommended, and earlier supplementation with artificial milk may be considered.

## Background

Almost all newborns lose weight in the first days of life [1, 2], and this is called physiological weight loss [3]. It is mainly due to fluid reduction [4]. Late cord clamping is related to a higher birth weight, which results in a greater weight change [5]. Neonatal weight loss is also a consequence of the use of adipose tissue as a source of energy by the newborns [3]. Excessive weight loss is associated with complications such as jaundice [6–9], hypoglycemia [10], and dehydration, which cause renal failure, thrombosis, hypovolemic shock, and seizures [11–19].

Breastfeeding undoubtedly provides health advantages to both the infant and the mother. Supplementation with formula should be avoided unless medically indicated as it may discourage the initiation and decrease the duration of breastfeeding [20–22]. The extent of a newborn’s weight loss in the first days of life is used as an indicator of breastfeeding adequacy [23]. The percentage of weight reduction that indicates formula supplementation is controversial [24]. A weight reduction of 10% is frequently applied because it is related to hypernatremic dehydration [25–27]. It is important to determine the risk factors for excessive weight loss to discover preventive measures.

Caesarean section is a known risk factor for excessive weight loss among newborns [1, 28]. However, little is known about the differences in risk according to the type of Caesarean section (antepartum or intrapartum) and the existence of other risk factors, especially in newborns at an accredited Baby-Friendly Hospital. The risk factors identified in the literature differ, depending on the setting [28–33]. Therefore, the aim of this study was to determine the factors associated with excessive weight loss in breastfed full-term newborns at a Baby-Friendly Hospital.

## Methods

### Study design, population, and setting

This was a retrospective observational study with no patient contact. It was conducted at a tertiary perinatal center (The National Hospital Organization Nagasaki Medical Center) in Japan. We included live babies who were born at ≥37 weeks of gestation between January 1, 2018, and December 31, 2018. We excluded cases of multiple births, cases in which babies were admitted to the neonatal intensive care unit (NICU) or referred to other facilities, and cases of exclusive formula feeding.

Our hospital is accredited as a Baby-Friendly Hospital and promotes the initiation of exclusive breastfeeding [34]. A total of 66 facilities, including 27 perinatal centers, are accredited as Baby-Friendly Hospitals in Japan [35]. In our hospital, early cord clamping is performed after a baby is delivered. In addition, we adhere closely to the “Ten Steps to Successful Breastfeeding” [34]. We do not have any lactation consultants. However, trained midwives or nurses help mothers after delivery to assure adequate breastfeeding. Temporary formula feeding is considered in cases of weight loss ≥10% in relation to the birth weight after a physical examination and blood tests, including the blood levels of glucose, urea nitrogen, and electrolytes, are performed. However, in cases of medical needs such as constant hypoglycemia or dehydration, minimal water or formula is given to infants even before weight loss reaches 10%. Hospital discharge at our facility routinely occurs when the infants are 5 days old in the case of vaginal delivery or 6 days old after a Caesarean section. All newborns are weighed daily between birth and discharge while naked with an electronic scale by a nurse or a midwife. Discharge is postponed if there are any maternal or neonatal complications, and discharge is not permitted until the infant’s weight begins to increase after its nadir.

### Data collection and definitions

We retrospectively obtained the demographic and perinatal data from the patients’ medical records. The following maternal and neonatal variables were assessed: maternal age, prepregnancy weight, prepregnancy body mass index (BMI), parity, infertility treatment, maternal weight gain during pregnancy, hypertension, diabetes, gestational week at delivery, use of magnesium sulfate in labor, use of oxytocin in labor, mode of delivery (vaginal delivery, intrapartum Caesarean section, or antepartum Caesarean section), postpartum hemorrhaging, maternal hemoglobin after delivery, birth weight, newborn sex, use of infant formula or water before a weight loss of ≥10% and neonatal jaundice requiring phototherapy. Prepregnancy BMI was calculated with maternal height at the first antenatal visit and self-reported prepregnancy weight. Maternal hypertension was defined as a systolic blood pressure ≥ 140 mmHg or a diastolic blood pressure ≥ 90 mmHg, regardless of the time of diagnosis, including before pregnancy, in pregnancy, and during the hospital stay. It included chronic hypertension, gestational hypertension, preeclampsia, and eclampsia. Diabetes included type 1, type 2, and gestational diabetes. Operative deliveries were classified as ‘antepartum’ if they were performed before the onset of labor and ‘intrapartum’ if they were performed after labor began [36]. The start of labor was defined as regular contractions occurring < 10 min apart and progressive cervical dilation or effacement [37]. All elective Caesarean sections were categorized as antepartum Caesarean sections. Emergency Caesarean sections could be antepartum or intrapartum Caesarean sections. Postpartum hemorrhaging was defined as a cumulative blood loss of ≥1000 mL within 24 h of the birth process, regardless of the mode of delivery [38]. Hemoglobin levels were measured within 2–3 days after delivery.

### Outcomes

The outcome was excessive weight loss, which was defined as a weight reduction of ≥10% in the first 5 days of life in relation to the birth weight.

### Secondary analysis

We examined whether there were any newborns who required intravenous therapy due to hypoglycemia or dehydration. We also investigated the association between neonatal jaundice requiring phototherapy and a higher weight loss.

We analyzed the blood test results of newborns who experienced a weight reduction of ≥10%. The plasma osmolality was calculated by the following formula: plasma osmolality = (sodium [mEq/L] × 2) + (glucose [mg/dL] / 18) + (blood urea nitrogen [mg/dL] / 2.8). The mean plasma osmolality results were compared among the groups stratified according to the mode of delivery.

### Statistical analysis

Data were entered in the Microsoft Excel software program (version 14.1.0; Microsoft®, Redmond, WA, USA) and exported to EZR (version 3.1.2; Saitama Medical Center, Jichi Medical University, Saitama, Japan), which was used to carry out the statistical analyses. Descriptive statistics were performed to determine the means (± standard deviations) and percentages. The Kolmogorov–Smirnov test was used to examine normality. The Student’s *t*-test or the Mann–Whitney U test was used to analyze 2 continuous variables, as appropriate. The ANOVA test or the Kruskal-Wallis test was used to analyze 3 continuous variables, as appropriate. Pearson’s chi-squared test was used to analyze categorical variables. Univariate logistic regression analysis of these variables was performed to estimate the crude odds ratios and their 95% confidence intervals (CIs). Then, we conducted multivariate logistic regression analysis. Factors with *p* values < 0.05 according to the univariate analysis were entered into the multivariate analysis. Neonatal jaundice requiring phototherapy was not included in the regression model of the primary outcome because neonatal jaundice is not the cause but the result of weight loss. Statistical interaction was examined between parity and the mode of delivery. *P* values < 0.05 were considered to indicate statistical significance.

### Ethical considerations

This study was approved by the Research Ethics Committee of the National Hospital Organization Nagasaki Medical under protocol number 2019079, approved on October 10, 2019, with opt-out consent to obtain patient data from the medical records. No ethical issues arose during this study as it was retrospective and all data were anonymous.

## Results

**Fig. 1:**
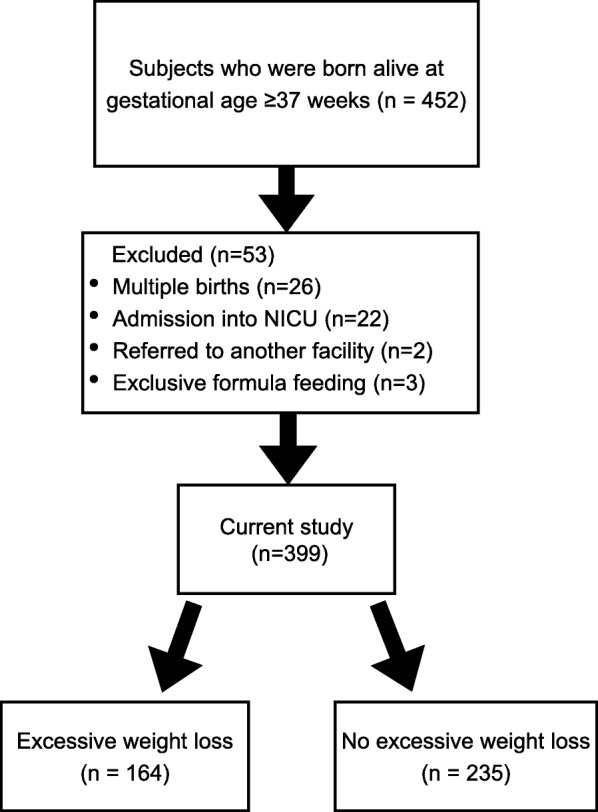
Flow chart of the patients included in this study

A total of 452 babies were born alive with a gestational age of ≥37 weeks during the study period, and 399 newborns were included in the current study after excluding 53 cases: 26 for multiple births, 22 for admission to the NICU, 2 for referral to another facility, and 3 for exclusive formula feeding. The mean percentage of weight reduction was 9.4%. Of these, 164 newborns (41%) lost ≥10% of their body weight (Fig. 1). The weight reached its nadir at day 3.0 ± 0.8 (mean ± standard deviation).

Table 1 shows the comparison of the maternal and neonatal characteristics between newborns with and without excessive weight loss. The mothers of newborns with excessive weight loss were older (*p* = 0.004), had a higher BMI (*p* = 0.043), were more frequently primiparous (*p* = 0.001), had more frequently received infertility treatment (*p* = 0.009), had more frequent postpartum hemorrhaging ≥1000 mL (*p* = 0.013), and had a lower hemoglobin level after delivery (*p* = 0.013) than mothers of newborns without excessive weight loss (Table 1).

**Table 1:** Comparison of the maternal and neonatal characteristics between newborns with and without excessive weight loss

	Weight loss ≥10% *N* = 164	Weight loss < 10% *N* = 235	Test value or U value	Degree of freedom	*p*
Maternal age, years^a^			15,989	NA	0.004
Minimum	21	16			
25 percentile	31	28			
Median	35	32			
75 percentile	37	37			
Maximum	45	43			
Prepregnancy maternal weight^a^			17,068	NA	
Minimum	35	40			
25 percentile	50	48			
Median	55	53			
75 percentile	63	60			
Maximum	105	111			
Prepregnancy BMI, kg/m^2a^			16,974	NA	0.043
Minimum	15.1	14.7			
25 percentile	19.8	19.2			
Median	22.0	21.2			
75 percentile	25.2	24.0			
Maximum	39.3	42.9			
Primiparity^b^	85 (52%)	82 (35%)	10.7	1	0.001
Infertility treatment^b^	30 (18.3%)	21 (8.9%)	6.8	1	0.009
Maternal weight gain, kg^a^			20,860	NA	0.16
Minimum	−4.0	−8.4			
25 percentile	6.3	6.6			
Median	8.7	9.5			
75 percentile	11.4	12.1			
Maximum	24.5	25.6			
Hypertension^b^	14 (8.5%)	14 (6.0%)	0.63	1	0.43
Diabetes^b^	42 (26%)	45 (19%)	2.0	1	0.16
Use of MgSO_4_ in labor^b^	2 (1.2%)	7 (3.0%)	0.68	1	0.41
Use of oxytocin in labor^b^	52 (32%)	59 (25%)	1.8	1	0.18
Gestational weeks at delivery^a^			18,431	NA	0.46
Minimum	37.1	37.0			
25 percentile	38.7	38.4			
Median	39.3	39.1			
75 percentile	39.9	40.0			
Maximum	42.1	41.7			
Mode of delivery^b^			5.9	2	0.052
Vaginal delivery	106 (64.6%)	178 (75.7%)			
Intrapartum CS	20 (12.2%)	21 (8.9%)			
Antepartum CS	38 (23.2%)	36 (15.3%)			
Postpartum hemorrhaging^b^	40 (24%)	33 (14%)	1.6	1	0.013
Hemoglobin after delivery, g/dL^a^			22,098	NA	0.013
Minimum	5.9	5.5			
25 percentile	9.3	9.6			
Median	10.1	10.4			
75 percentile	11.0	11.2			
Maximum	12.6	13.6			
Birth weight of infant < 2500 g^b^	10 (6.1%)	23 (9.8%)	1.3	1	0.26
Male^b^	80 (49%)	113 (48%)	0.0012	1	0.97
Use of infant formula or water before weight loss ≥10%^b^	22 (13%)	33 (14%)	0.001	1	0.98
Neonatal jaundice requiring phototherapy^b^	22 (13%)	19 (8.1%)	2.4	1	0.12

Abbreviations: *BMI* body mass index, *CS* Caesarean section, *NA* not applicable

^a^ The Mann–Whitney U test was used

^b^ The Pearson’s chi-squared test was used

Table 2 shows the univariate and multivariate logistic analysis results of the associations between the variables and excessive weight loss in newborns. According to the univariate analysis, maternal age, primiparity, infertility treatment, antepartum Caesarean section, postpartum hemorrhaging, and the hemoglobin level after delivery were associated with excessive weight loss in newborns. Meanwhile, according to the adjusted multiple regression analysis, the factors associated with excessive weight loss in newborns were an older maternal age, primiparity, and antepartum Caesarean section, with adjusted odds ratios (95% Confidence Intervals [CIs]) of 1.07 (1.02, 1.11), 2.72 (1.69, 4.38), and 2.00 (1.09, 3.65), respectively (Table 2). No statistical interaction was noted between parity and the mode of delivery, or between maternal age and infertility treatment.

**Table 2:** Multivariate logistic analysis of the association between the variables and excessive weight loss

	Crude OR (95% CI)	*p*	Adjusted^a^ OR (95% CI)	*p*
Maternal age	1.06 (1.02, 1.10)	0.001	1.07 (1.02, 1.11)	0.002
Prepregnancy BMI, kg/m^2^	1.04 (0.99, 1.09)	0.13	1.04 (0.99, 1.09)	0.15
Primiparity	2.01 (1.34, 3.02)	< 0.001	2.72 (1.69, 4.38)	< 0.001
Infertility treatment	2.28 (1.25, 4.15)	0.007	1.27 (0.65, 2.52)	0.49
Maternal weight gain, kg	0.99 (0.95, 1.03)	0.58	1.00 (0.96, 1.05)	0.91
Hypertension	1.47 (0.68, 3.18)	0.32	1.15 (0.51, 2.59)	0.74
Diabetes	1.45 (0.90, 2.34)	0.14	1.40 (0.83, 2.35)	0.21
Use of MgSO_4_ in labor	0.40 (0.08, 1.96)	0.26	0.28 (0.06, 1.42)	0.13
Use of oxytocin in labor	1.38 (0.89, 2.15)	0.15	1.13 (0.68, 1.88)	0.65
Gestational weeks at delivery	1.05 (0.86, 1.28)	0.62	1.09 (0.87, 1.36)	0.47
Mode of delivery				
Vaginal delivery	1		1	
intrapartum CS	1.60 (0.83, 3.09)	0.16	0.99 (0.48, 2.05)	0.98
antepartum CS	1.77 (1.06, 3.00)	0.030	2.00 (1.09, 3.65)	0.024
Postpartum hemorrhaging	1.97 (1.18, 3.30)	0.009	1.31 (0.71, 2.41)	0.39
Hemoglobin after delivery, g/dL	0.83 (0.71, 0.97)	0.019	0.93 (0.78, 1.12)	0.45
Birth weight of infant < 2500 g	0.60 (0.28, 1.29)	0.19	0.58 (0.26, 1.32)	0.19
Male	1.03 (0.69, 1.53)	0.83	1.05 (0.69, 1.60)	0.82
Use of infant formula or water before weight loss ≥10%	0.95 (0.53, 1.69)	0.86	0.65 (0.34, 1.23)	0.18

Abbreviations: *BMI* body mass index, *CS* Caesarean section

^a^ Adjusted for maternal age, primiparity, infertility treatment, antepartum Caesarean section, postpartum hemorrhaging, and the hemoglobin level after delivery

No full-term newborn included in this study required intravenous therapy due to hypoglycemia or dehydration. However, neonatal jaundice requiring phototherapy was associated with a higher weight loss (percentage) (odds ratio: 1.22; 95% CI: 1.05–1.42).

Table 3 shows the laboratory test results of the newborns who were examined on the day of weight loss ≥10%. The mean plasma osmolalities on the day of weight loss ≥10% were 306, 304, and 303 mOsm/kgH_2_O in newborns delivered by vaginal delivery, intrapartum Caesarean section, and antepartum Caesarean section, respectively (*p* = 0.043).

**Table 3:** Comparison of the blood test results according to the mode of delivery

	Vaginal delivery*N* = 106	Intrapartum Cesarean section*N* = 20	Antepartum Cesarean section*N* = 38	Test value or F value	Degree of freedom	*p*
Sodium, mEq/L^a^				0.96	2	0.62
Minimum	142	141	143			
25 percentile	147	146.8	147			
Median	148	148	148			
75 percentile	150	149.5	150			
Maximum	154	152	154			
Potassium, mEq/L^a^				6.5	2	0.038
Minimum	3.2	3.8	3.4			
25 percentile	4	4.1	3.8			
Median	4.3	4.3	4.1			
75 percentile	4.7	4.7	4.3			
Maximum	7.1	5.4	5.7			
Ionized calcium, mmol/L^b^	1.21 ± 0.08	1.17 ± 0.07	1.21 ± 0.08	2.7	2	0.069
Glucose, mg/dL^a^				0.29	2	0.87
Minimum	31	39	34			
25 percentile	45	46	47			
Median	53	53	51			
75 percentile	65	58	60			
Maximum	88	84	90			
Blood urea nitrogen, mg/dL^a^				18.4	2	< 0.001
Minimum	3	5	3			
25 percentile	11	12	7			
Median	15	14	9			
75 percentile	19	18	12			
Maximum	37	31	22			
Creatinine, mg/dL^a^				3.3	2	0.19
Minimum	0.3	0.3	0.4			
25 percentile	0.6	0.6	0.5			
Median	0.7	0.8	0.6			
75 percentile	0.8	0.8	0.7			
Maximum	1.3	1.2	1.1			
Osmotic pressure as osmolality, mOsm/kgH2O^b^	305.7 ± 5.8	304.2 ± 4.8	303.0 ± 5.3	3.2	2	0.043

^a^ The Kruskal-Wallis test was used

^b^ The ANOVA test was used

## Discussion

Our data showed a high mean percentage of weight reduction (9.4%) and a high rate (41%) of excessive weight loss in healthy full-term newborns at our center. The mean percentage of weight reduction in our center was much higher than that reported in the literature in other developed countries (2.4–8.6%) [25–27, 31, 39, 40]. This discrepancy may be partly due to the delayed lactogenesis in Asians compared to other ethnic groups [41]. It is also because we check the weight every day and keep a baby in the hospital until we confirm a nadir of the weight. The rate is also higher than that of other perinatal centers accredited as Baby-Friendly Hospitals in Japan. In 2018, the mean weight reduction rates at 27 perinatal centers accredited as Baby-Friendly Hospitals in Japan were 8.2 and 8.9% for vaginal delivery and Caesarean section, respectively [42]. In comparison, the rates were 9.0 and 9.9% at our center, respectively [42]. Our cohort’s baseline characteristics were similar to those of 27 perinatal centers accredited as Baby-Friendly Hospitals in Japan [42]. This difference of the weight reduction is possibly related to the high rate of exclusively breastfed newborns. The same report showed that the mean rate of exclusively breastfed newborns at hospital discharge at 27 perinatal centers was 77% [42], while the rate at our center was 84% [42].

An older maternal age [29] and primiparity [19, 30] also have been shown as risk factors for excessive newborn weight loss in previous studies [29, 30]. An older maternal age [43, 44] and primiparity [30, 45, 46] are associated with excessive weight loss in newborns due to the delayed onset of lactation.

The most notable finding of this study was that there was a significant difference in the frequency of excessive weight loss between the newborns delivered by antepartum Caesarean section and those delivered by vaginal delivery, while no marked difference was noted between infants delivered via intrapartum Caesarean section and those delivered by vaginal delivery. Most of the previous studies on this topic did not distinguish between antepartum Caesarean section and intrapartum Caesarean section. Our finding is consistent with one previous study [47]. In addition, an observational cohort study has demonstrated that primary antepartum Caesarean section is an independent risk factor for the failure of exclusive breastfeeding [48]. However, their study outcome was different from ours, and the study did not distinguish between the experience of never trying to breastfeed and that of trying but not succeeding.

Three hypotheses may explain these findings. First, labor causes hormonal changes in the mother, and these changes promote lactogenesis. Some studies suggest that labor can elevate the plasma concentrations of oxytocin and prolactin [49, 50]. Second, labor causes hormonal changes in newborns, and these changes affect the kidney function and insensible water loss. For example, glucocorticoids are known to reduce the rate of insensible water loss in newborns [51], and oxytocin is an antidiuretic [52]. Third, labor itself or the excessive administration of fluids to the mother before she gives birth may affect the amount of total body water in the newborn at birth. An observational cohort study has shown that the timing and the amount of maternal intravenous fluids are related to the neonatal output and newborn weight loss [53]. The differences of mean plasma osmolality on the day of ≥10% in newborns delivered by vaginal delivery, intrapartum Caesarean section, and antepartum Caesarean section indicated that the newborns delivered by antepartum Caesarean section tended to be born with more body water, and the shedding of this excess water after birth accounts for part of the weight loss. Further research is necessary to determine the mechanisms involved.

The results of the present study suggest that earlier intervention including closer monitoring of mothers and babies as well as earlier initiation of formula feeding in cases with an older maternal age, primiparity, and antepartum Caesarean section may help to prevent complications in newborns due to excessive weight loss.

Several limitations associated with the present study warrant mention. First, because our study population was derived from one hospital in Japan, the generalizability of our study results might be limited to some extent. Second, there might be other factors associated with excessive weight loss in newborns. For example, we did not examine the rate of weight loss or the feeding methods in previous pregnancies. In addition, we did not analyze drug use in labor or pregnancy except for oxytocin and magnesium sulfate use in labor, which might affect infant feeding. Third, prepregnancy BMI is subject to memory bias and the timing of weight measurement is imprecise. Fourth, the accurate amount of formula or water administered before weight loss reaches 10% was not documented. However this had little effect on weight loss because minimal amount was usually given in a single dose.

## Conclusions

An older maternal age, primiparity, and antepartum Caesarean section were found to be independent risk factors for excessive weight loss in breastfed full-term newborns in this study. Closer monitoring of these infants is recommended and earlier supplementation with artificial milk may be indicated.

## Data Availability

The datasets used and analyzed during the current study are available from the corresponding author on reasonable request.

## Abbreviations

BMI: Body mass index
CI: Confidence interval
NICU: Neonatal intensive care unit
